# Reactions of the Antiarthritic Drug Aurothiomalate With Phenylmercury(II) Compounds: NMR Studies

**DOI:** 10.1155/MBD.1996.269

**Published:** 1996

**Authors:** Garry G. Graham, Diarmaid Gordon, Peter J. Sadler

**Affiliations:** 1 School of Physiology and Pharmacology University of NSW Sydney NSW 2052 Australia; 2 Department of Chemistry Birkbeck College University of London Gordon House and Christopher Ingold Laboratory, 29 Gordon Square London WC1H OPP United Kingdom

## Abstract

Clinical formulations of the antiarthritic drug aurothiomalate sometimes contain phenylmercury(ll) compounds as antimicrobial preservative agents and, in the presence of *para*-chloromercuri-benzoate, aurothiomalate is a potent inhibitor of collagenase. By ^1^H NMR spectroscopy, *para*-hydroxymercuribenzoate and *para*-hydroxymercuriphenylsulphonate were shown to react with aurothiomalate by complexing only with the terminal thiomalate of aurothiomalate oligomers, thereby converting them to ring complexes. The reaction was also detected by UV spectroscopy. The arylmercury complexes produced no change in the bulk of the thiomalate residues of aurothiomalate indicating considerable stability of the polymeric structure of aurothiomalate in which each gold is bound to two thiolate residues. The potent inhibition of the mercurial induced collagenase activity may be due either to aurothiomalate or to a complex formed between the terminal thiomalate residues with the arylmercurial. The arylmercury complexes may be unsuitable as antimicrobials in solutions of aurothiomalate because of complexation with the terminal thiomalate residues.


### REACTIONS OF THE ANTIARTHRITIC DRUG AUROTHIOMALATE WITH PHENYLMERCURY(II) COMPOUNDS: NMR STUDIES

Garry G. Graham[1], Diarmaid Gordon[1] and Peter J. Sadler*[2]

[1]School of Physiology and Pharmacology, University of NSW, Sydney, NSW 2052, Australia
[2]Department of Chemistry, Birkbeck College, University of London, Gordon House and Christopher Ingold Laboratory, 29 Gordon Square, London WC1H OPP, UK

**ABSTRACT** Clinical formulations of the antiarthritic drug aurothiomalate sometimes contain phenylmercury(II) compounds as antimicrobial preservative agents and, in the presence of *para*-chloromercuri-benzoate, aurothiomalate is a potent inhibitor of collagenase. By $^1H$ NMR spectroscopy, *para*-hydroxymercuribenzoate and *para*-hydroxymercuriphenylsulphonate were shown to react with aurothiomalate by complexing only with the terminal thiomalate of aurothiomalate oligomers, thereby converting them to ring complexes. The reaction was also detected by UV spectroscopy. The arylmercury complexes produced no change in the bulk of the thiomalate residues of aurothiomalate indicating considerable stability of the polymeric structure of aurothiomalate in which each gold is bound to two thiolate residues. The potent inhibition of the mercurial induced collagenase activity may be due either to aurothiomalate or to a complex formed between the terminal thiomalate residues with the arylmercurial. The arylmercury complexes may be unsuitable as antimicrobials in solutions of aurothiomalate because of complexation with the terminal thiomalate residues.

### INTRODUCTION

Aurothiomalate ("Myocrisin") is a widely used antiarthritic drug. It has never been crystallized, but X-ray absorption fine structure analysis and wide angle X-ray scattering measurements suggest that it contains hexameric rings and oligomeric chains, consistent with the presence of about 10-15% excess of thiomalate over gold in most preparations [1,2] (Fig. 1). We report here studies of the interaction of aurothiomalate with phenylmercury(II) complexes by NMR and UV spectroscopy. Interest in the interaction of aurothiomalate with arylmercury(II) complexes arises firstly from the observation that aurothiomalate is a potent inhibitor of the enzyme collagenase ($IC_{50}$ 3.5 nM [3]) in the presence of *para*-hydroxymercuribenzoate (BHgOH) which activates the enzyme [3,4]. Aurothiomalate alone is only a weak inhibitor of collagenase when isolated in its active form [3,5] and a weak activator of the latent enzyme [3]. Secondly, an arylmercurial, phenylmercury(II) nitrate, has been used as a bactericide in some formulations of aurothiomalate. The presence of an arylmercurial which can bind thiolate groups in a preparation of a drug which is itself a thiolate complex allows the possibility of complex chemical interactions. It is clearly important to know the precise chemical composition of any preparation which is administered to man.

Arylmercury(II) complexes, particularly BHgOH, are widely used in biochemistry as thiol reagents and the nature of their reactions with the free ligand, thiomalate, is of general interest in biochemistry. In the present work, the chemical reactions between aurothiomalate and thiomalate with arylmercury(II) complexes were studied mainly using *para*-hydroxymercuriphenylsulphonate (PSHgOH) as the prototype arylmercury(II) complex. It is highly soluble in water and can be buffered to neutral pH values without precipitation. By comparison, BHgOH is difficult to use because it precipitates as the free acid at pH values below about 9 but it was used in unbuffered solutions to confirm the findings produced with PSHgOH. Phenylmercuric nitrate is sparingly soluble in water (about 1 mM) and therefore BHgOH and PSHgOH were preferred for the NMR work described here.

Although arylmercurials are widely used as thiol reagents, there is surprisingly little detailed information on the nature of their reactions with thiols, particularly using modern techniques. Spectrophotometric titrations of BHgOH with thiomalate show a gradual increase in absorption at about 250 nm with increasing amounts of the mercurial with a sharp end-point consistent with a single product of the type, BHgStm, although the corresponding titration with cysteine indicated the possibility of more than one organomercurial reacting with the cysteine when BHgOH was in excess [6]. Data obtained in the present study indicate more complex interactions between arylmercuric complexes than previously detected.

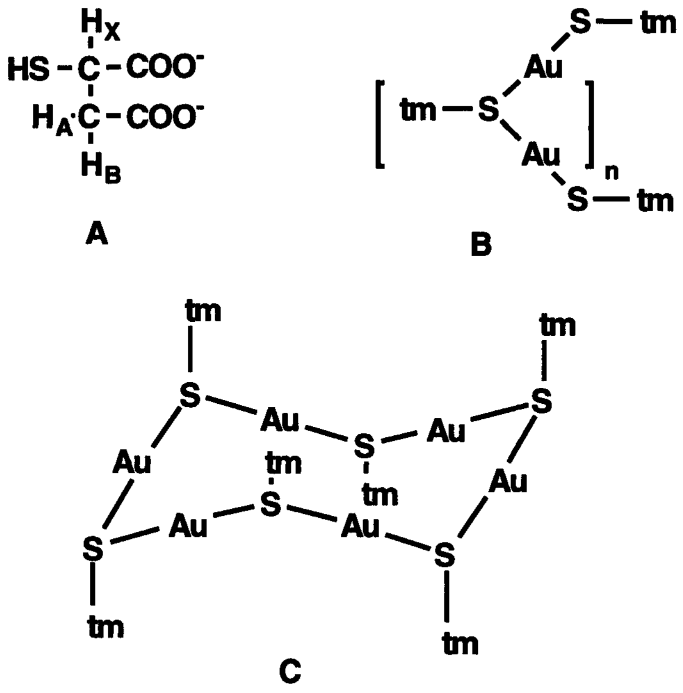

**Figure 1.** (A) Structure of thiomalate showing protons giving rise to the ABX $^1$H NMR spectrum. (B) Oligomeric structure of aurothiomalate. Note that the chain is terminated by thiomalate residues and that there must be one more thiomalate residue than gold atoms in the oligomer.
(C) Hexameric ring structure of aurothiomalate.

### MATERIALS AND METHODS

Aurothiomalate, the kind gift of Rhône Poulenc (Dagenham), was an off-white solid with approximate composition $Au(tm)_{1.15}$. 0.3(glycerol). $2H_2O$. Thiomalic acid and the sodium salts of PSHgOH, and BHgOH were purchased from Sigma. Solutions of sodium thiomalate were prepared by neutralization of thiomalic acid solutions with sodium hydroxide and, for NMR spectroscopy, were then freeze-dried twice from $D_2O$. Reactions between aurothiomalate, thiomalate and the mercury complexes were studied by NMR in $D_2O$ solutions. Reactions involving BHgOH were carried out in unbuffered solutions with a final pH* (pH meter reading in $D_2O$) of about 9.2; attempts to lower the pH* resulted in precipitation. In addition, the reactions of aurothiomalate and thiomalate with PSHgOH were carried out in 0.1 M phosphate buffer in $D_2O$, pH* 7.0. The final pH* values ranged from 6.99 to 7.03.

$^1H$ NMR spectra were recorded on Varian VXR400 and JEOL GSX500 spectrometers at 400 and 500 MHz respectively, and $^{199}Hg$-{$^1H$} NMR spectra at 71.3 MHz on the Varian instrument. For $^1H$ NMR, 5 mm tubes were used and peaks referenced to sodium trimethylsilyl-$d_4$-propanoate. Typical pulse conditions were 5000 Hz spectral width, 32 K computer points, 3.3 s acquisition time, 45° pulses, 128 scans. For $^{199}Hg$-{$^1H$} spectra, 10 mm tubes were used and typical pulse conditions were 100 KHz spectral width, 32 K computer points, 0.16 s acquisition time, 90° pulses, 0.5 s pulse delay, and 8000 scans. Mercuric chloride (1 M) in ethanol was used as an external standard.

UV spectra were obtained on a Hitachi spectrophotometer (model U-3210) with 1 cm cells at room temperature. Aurothiomalate (100 to 300 μM) or thiomalate (10 to 50 μM) were mixed with PSHgOH (50 μM) in 0.05 M phosphate buffer, pH 7.0. Absorbances at 240, 245, 250 and 260 nm were recorded and the absorbances due to PSHgOH and aurothiomalate subtracted. Solutions of aurothiomalate were allowed to stand for at least 2 hours before the addition of PSHgOH to avoid absorbance changes which occur on dissolution [7].

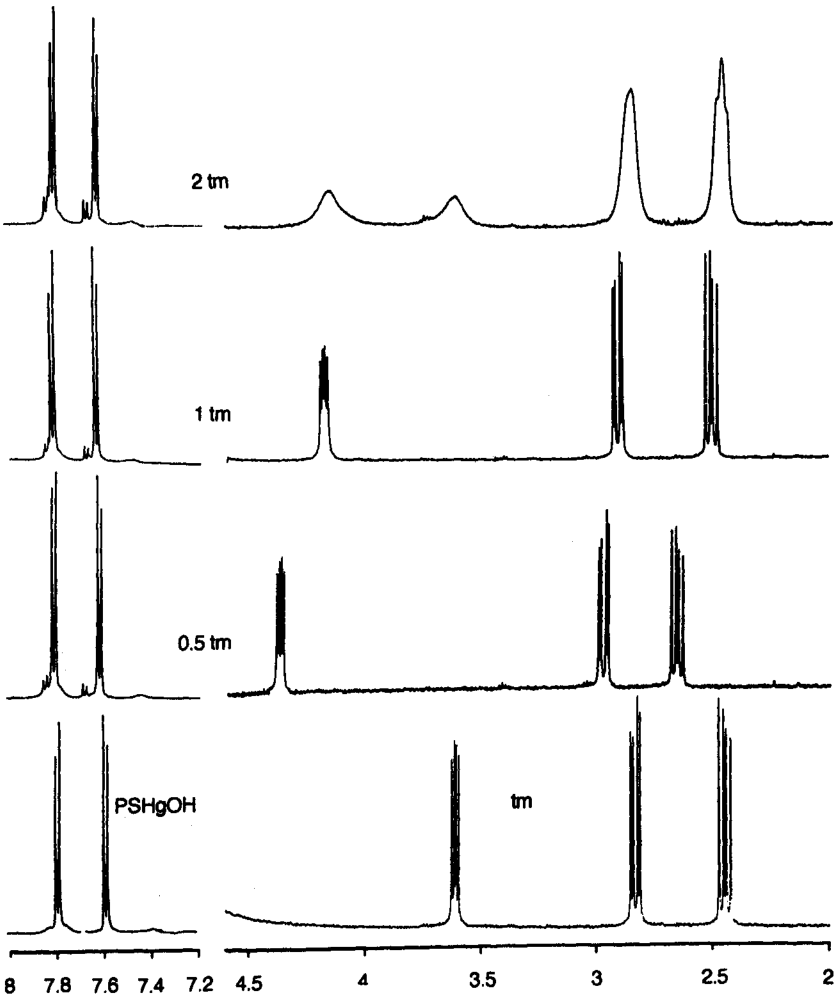

**Figure 2.** Aromatic (left) and aliphatic (right) regions of 500 MHz $^1H$ NMR spectra of thiomalate and PSHgOH alone, and at mol ratios of 0.5:1, 1:1, and 2:1.

### RESULTS

First we studied the reaction between thiomalate and BHgOH and PSHgOH by $^1$H NMR spectroscopy. Thiomalate itself exhibits an ABX-type spectrum with the $CH_x$ proton downfield of the peaks from the non-equivalent $CH_2$ protons (Figs. 1,2) [8]. In this aliphatic region of the $^1$H NMR spectrum, the addition of PSHgOH produced a marked downfield shift in all resonances, being largest for the $CH_x$ resonance (Fig. 2, Table I).

This downfield shift is consistent with the deshielding of the protons of thiomalate on complexation with the organomercurial to give PSHgStm, where PSHg is the *para*-mercuri-phenylsulphonate group and Stm is the thiolate group in thiomalate. In the presence of excess thiomalate, broad resonances were seen indicating equilibration between free thiomalate and PSHgStm at an intermediate rate on the NMR time scale (Fig. 2). The downfield shifts were greatest when the mercurial was in excess, suggesting the formation of an intermediate, $(PSHg)_2Stm$.

Clear evidence of interaction between the PSHgOH and thiomalate was also shown by changes in the NMR spectra of the *ortho* and *meta* protons of the organomercurial. The two doublets in this region, and their $^{199}$Hg satellites shifted progressively downfield (Fig. 2).

The resonances of aurothiomalate formed three poorly defined groups with chemical shifts of approximately 2.68, 2.86 and 4.07 p.p.m and were considerably sharpened and shifted slightly upfield by the reaction with PSHgOH (Fig. 3). Thus, there has been little change in the chemical environment of the bulk of the thiomalate residues in aurothiomalate, the sharpening of the peaks being possibly due to the removal of chain terminating thiomalate and therefore the absence of line-broadening due to chemical exchange (Fig 1). Smaller peaks in the aliphatic region of the NMR spectra indicate the formation of adducts of PSHgOH with some of the thiomalate ligands present in aurothiomalate. In particular, the broad peak at 4.15 p.p.m. seen at a 5:1 mol ratio of aurothiomalate to the organomercurial is consistent with the formation of a thiomalate mercurial complex.

Clearer evidence for the reaction between PSHgOH and some of the thiomalate ligands in aurothiomalate is shown by the changes in the resonances in the aromatic region of the NMR spectra. At a 10:1 mol ratio of aurothiomalate to the mercury complex, the major resonances are centered at 7.63 and 7.83 ppm, identical with the chemical shifts of products of the interaction PSHgOH and thiomalate alone. Similar major resonances are seen in the 1:1 and 2:1 mixtures of thiomalate and PSHgOH when the major product of the reaction should be the monothiolato complex, PSHgStm (Fig. 3). At lower mol ratios of PSHgOH to aurothiomalate (5:1 and 2:1), there is considerable broadening of the major aromatic resonances, attributable to exchange of $PSHg^+$ between the different species, e.g. $(PSHg)_2Stm$, PSHgStm and PSHgOH at an intermediate rate on the NMR timescale. No such broadening was observed with thiomalate and excess PSHgOH indicating that exchange of $PSHg^+$ with free thiomalate is faster than with gold bound thiomalate.

Reaction between PSHgOH and some of the thiomalate residues of aurothiomalate was also evident from the appearance of two doublets in the aromatic region corresponding to the minor product of the reaction between the PSHgOH and thiomalate itself (Figs. 2 and 3). This product was detectable in all mixtures of aurothiomalate and PSHgOH at mol ratios from 0.5:1 to 5:1 but absent from the 10:1 mixture. When present, the NMR resonances from this minor product remained sharp with constant chemical shifts indicating that this species is not in rapid exchange with the other arylmercurial complexes present.

Reactions between thiomalate and aurothiomalate with BHgOH followed a similar pattern to those with PSHgOH although the reactions could not be studied at physiological pH values. The major difference was that no species corresponding to $(PSHg)_2Stm$ was seen in solutions of thiomalate and BHgOH. A minor product was also seen from the pairs of doublets in the aromatic region.

Reaction of thiomalate ligands in aurothiomalate solutions with PSHgOH was confirmed by ultraviolet spectroscopy. Increased absorbance in the range 240 to 260 nm resulted from the addition of PSHgOH to both thiomalate and aurothiomalate, indicating the production of a S-bound thiomalate arylmercuric complex (Fig. 4). Comparison of the absorbance change with that produced by the reaction of PSHgOH with thiomalate alone indicated that about 10% of the thiomalate in aurothiomalate reacted the arylmercuric complex. The accuracy of this procedure was limited by the high background absorbance of aurothiomalate of about ten times the absorbance due the arylmercuric thiomalate complex. Furthermore, the complexation of chain-terminating thiomalate groups and consequent changes in the structure of aurothiomalate may alter the absorbance of aurothiomalate.

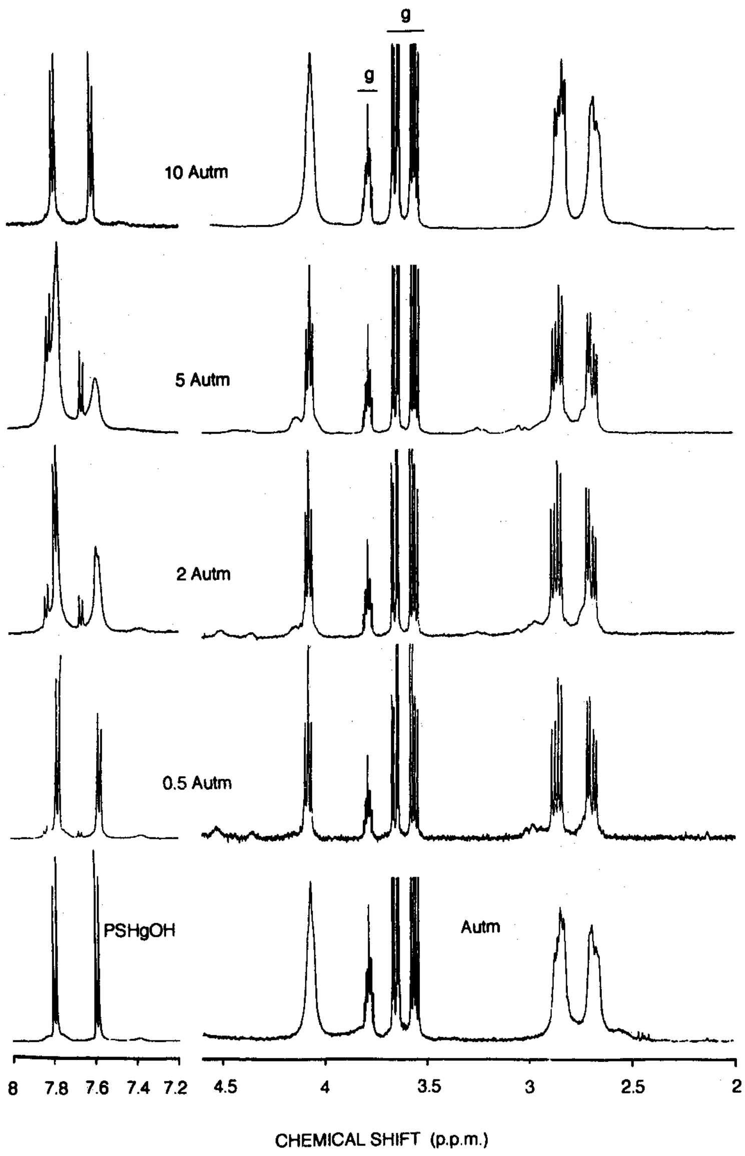

**Figure 3.** Aromatic (left) and aliphatic (right) regions of 500 MHz $^1H$ NMR spectra of aurothiomalate and PSHgOH alone, and at mol ratios of 0.5:1, 1:1, 2:1, 5:1 and 10:1. The resonances labelled g arise from glycerol which is present in sodium aurothiomalate.

**Table I.** Chemical shifts (parts per million) and coupling constants (Hz) of $^1$H NMR resonances of aurothiomalate (Autm), thiomalate (tmSH) and complexes formed by the interaction between thiomalate (tmSH) and PSHgOH.

| Species | Aliphatic resonances | | | | | | Aromatic resonances | | |
|---|---|---|---|---|---|---|---|---|---|
| | Chemical shift | | | Coupling constant | | | Chemical shift | | Coupling constant |
| | $CH_A$ | $CH_B$ | $CH_X$ | $J_{AB}$ | $J_{AX}$ | $J_{BX}$ | $CH_A$ | $CH_B$ | $J_{AB}$ |
| tmSH | 2.829 | 2.446 | 3.611 | 15.4 | 5.8 | 9.6 | | | |
| PSHgOH:tmSH (2:1) | 3.000 | 2.695 | 4.417 | 15.7 | 4.8 | 9.5 | 7.809 | 7.616 | 8.4 |
| PSHgOH:tmSH (1:1) | 2.901 | 2.501 | 4.165 | 15.0 | 5.1 | 9.9 | 7.820 | 7.637 | 8.2 |
| PSHgOH | | | | | | | 7.789 | 7.588 | 8.4 |
| Autm | 2.85* | 2.68* | 4.07* | | | | | | |

* Approximate chemical shifts

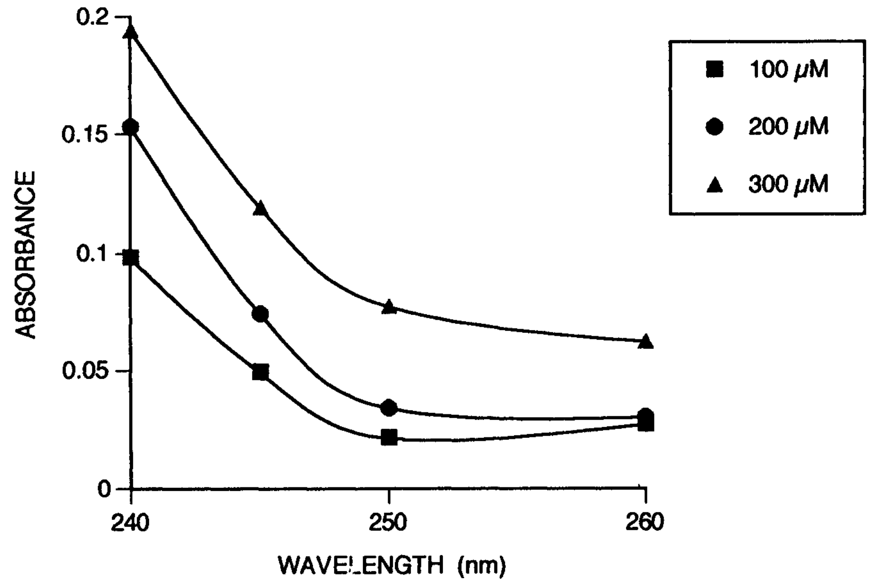

**Figure 4.** UV difference spectra of aurothiomalate (100 to 300 μM) in the presence of PSHgOH (50 μM) in phosphate buffer, pH 7.0. The recorded absorbances are those of mixtures of aurothiomalate and PSHgOH from which the absorbances of unreacted aurothiomalate and PSHgOH have been subtracted.

The reaction between PSHgOH and thiomalate was also studied by $^{199}Hg$ NMR. The chemical shifts of PSHgOH was -1365 p.p.m. increasing progressively to -1240 and -913.7 ppm at ratios of thiomalate to PSHgOH of O.5:1 and 1:1, respectively. No further change in the resonance of $^{199}Hg$ occurred on the addition of a further equivalent of thiomalate.

The nature of the PSHgOH complexes with thiomalate was also investigated by matrix assisted laser desorption mass spectrometry. A solution containing PSHgOH and thiomalate in a 1:1 mol ratio, however, did not give rise to any observable peaks.

### DISCUSSION

Arylmercury complexes are useful thiol reagents in biochemistry, but a simple 1:1 complex is not the only product formed. A bisarylmercury(II) complex of the type $(PSHg)_2Stm$ may be formed in the presence of excess arylmercurial mercurial complex and a minor product, possibly $PSHg(Stm)_2$, is formed in the presence of excess thiomalate. The latter type of complex in which the coordination number of mercury is 3 has been reported with methylmercury although the stability constant is predicted to be low [9]. It is probable that the stabilities of similar complexes of arylmercury complexes are also low.

As discussed above, aurothiomalate forms chains which are terminated by the excess thiomalate present and the interaction of aurothiomalate and PSHgOH is consistent with the reaction of only this excess thiomalate with the organomercurial. The corollary, however, is that the chains must lengthen if the gold in aurothiomalate remains bound to two sulphur atoms or that cyclic structures are formed (Fig. 1). Despite this possible lengthening of the aurothiomalate polymer, the NMR resonances of the thiomalate resonances actually sharpened on the addition of the arylmercurials. Similar sharpening of the broad thiomalate resonances in aurothiomalate was noted when small amounts of the thiol reagent, 5,5'-dithiobis-(2-nitrobenzoic acid) (Ellman's reagent) were added to aurothiomalate [10]. Possibly, the removal of chain terminating thiolates leads to the production of cyclic structures with sharper NMR resonances due to the removal of exchange broadening reactions (Figs. 1 and 2).

The conclusion that the structure of aurothiomalate is not greatly altered by the arylmercurials is consistent with previous data demonstrating that the reactivity of aurothiomalate towards cysteine is also not altered by the presence of aryl mercurials. The latter was indicated by the data of Danpure and Fyfe [11] who found that the aryl mercurials do not inhibit the interaction between aurothiomalate and cysteine although the interaction between cysteine and the aryl mercurials is blocked. The complex formed between PSHgOH and thiomalate is very stable (log association constant 17.3) [12], and. the lack of interaction of PSHgOH with the bulk of the thiomalate residues in aurothiomalate is consistent with the very considerable stability of the Au - thiolate bond and the polymeric structure of aurothiomalate.

There has been little published work on the NMR spectroscopy of the aryl mercurial complexes. It was reported that the chemical shifts of the ortho protons of various PhHgX compounds (where Ph = phenyl) in dimethylsulphoxide are independent of the nature of the X group whereas the magnitudes of the coupling constants, J($^1$Hortho - $^{199}$Hg), vary with the substituents [13]. A similar result was found in the present study. The resonances of both the *ortho* and *meta* protons of the arylmercurials were shifted only slightly downfield by the reactions of the arylmercurials with thiomalate (Fig 2).

Apart from giving basic information on the chemistry of aurothiomalate, this work was also designed to give information on questions about the interaction between aurothiomalate and arylmercury complexes. The first concerned the inhibitory action of aurothiomalate on collagenase activated by BHgOH. In this system, the inhibitory effect of aurothiomalate was shown at concentrations far below the concentrations of the arylmercurial [3]. Such conditions could not be reproduced in the present NMR spectroscopic studies but from the present studies where there was an excess of the mercurial, it seems unlikely that the basic structure of aurothiomalate should be altered. It is probable that BHgOH forms complexes only with the chain terminating thiomalate residues and, if free BHgOH is available after its activation of collagenase, the total concentration of complexes of BHgOH with thiomalate should be about 10 to 15% of that of aurothiomalate. Thus, solutions of BHgOH and excess aurothiomalate should contain a cyclised form of aurothiomalate and a thiomalato complex with BHgOH. Although the concentration of the thiomalate-arylmercury complex should be lower than that of aurothiomalate, it is not possible to predict which species is inhibiting the collagenase.

The present studies also provide information about the nature of the general reaction between arylmercurials and thiol compounds as well as their interaction with aurothiomalate. Typically, the commercial ampoules have contained far higher concentrations of aurothiomalate (about 50 mM) compared to about 30 μM phenylmercury(II) nitrate as an antimicrobial agent. Under these conditions, it is probable that the products formed would resemble those in the 10:1 mixture of aurothiomalate: PSHgOH. i.e. the aurothiomalate should be very largely unaltered while the lower amount of the phenylmercury(II) nitrate should be completely converted to the thiolate derivative of the aryl mercury compound. With this chemical change, the anti-microbial activity of phenylmercury(II) nitrate would appear very doubtful. Recently, aurothiomalate solutions have been sterilized by filtration rather than by heating in the presence of phenylmercury(II) nitrate. This appears to be a useful change in view of the probable inactivity of the arylmercury complex in the presence of aurothiomalate.

### ACKNOWLEDGEMENTS

We thank the EPSRC, MRC, University of London Intercollegiate Research Service, and Wolfson Foundation for their support for this work. We are grateful to Mr D. Shipp and Dr H. G. Parkes for assistance with NMR measurements and for Mr R. Lidgard for help with the mass spectrometry.

### REFERENCES

1. Rudge S.R., Perrett D., Swannell A.J., Drury P.L. *J. Rheumatol* 11,150-152 (1984).
2. Grootveld, M.C., Razi, M.T., Sadler, P.J. *Clin. Rheumatol.* **3** (Suppl 1), 5-16 (1984).
3. Mallya, S.K., van Wart, H.E. *Biochem. Biophys. Res. Commun.* **144**, 101-108 (1987).
4. Mallya, S.K., van Wart, H.E. *J. Biol. Chem.* **264**, 1594-1601 (1989).
5. Lindy, S., Sorsa, T., Suomalainen, K., Turto, H. *FEBS Letters* **208**, 23-25 (1986).
6. Boyer, P.D. *J. Am. Chem. Soc.* **76**, 4331-4337 (1954).
7. Grootveld, M.C., Sadler, P.J. *J. Inorg. Biochem.* **19**, 51-64 (1983).
8. Isab, A.A., Sadler, P.J. *J. Chem. Soc. Dalton* 1657-1663 (1981).
9. Schwarzenbach G., Schellenberg M. *Helv Chim Acta* **48**,28-46 (1965).
10. Reglinski, J., Hoey, S., Smith, W.E. *Inorg. Chim. Acta* **152**, 261-264 (1988).
11. Danpure, C.J., Fyfe, D.A. *Biochem. Soc. Trans.* **4**, 631-633 (1976).
12. Rabenstein D.L., Bravo J. *Inorg Chem* **26**, 2784-2787 (1987).
13. Petrosyan, V.S., Reutov, O.A. J. *Organomet. Chem.* **76**, 123-169 (1974).

**Received: September 16, 1996 - Accepted: September 23, 1996 - Received in revised camera-ready format: October 14, 1996**

